# Congenital Unilateral Hypertrophy of the Plantar Musculature with Multiple Toe Deformities: A Case Report and Literature Review

**DOI:** 10.1155/2020/8402531

**Published:** 2020-12-24

**Authors:** Matthias Holzbauer, Stefan Rick, Marco Götze, Sébastien Hagmann

**Affiliations:** ^1^Johannes Kepler University Linz, Faculty of Medicine, Altenberger Strasse 69, 4040 Linz, Austria; ^2^maz-Microsurgical Training and Research Center, Kepler University Hospital GmbH, Altenberger Strasse 69, 4040 Linz, Austria; ^3^BG Trauma Center Ludwigshafen, Ludwig-Guttmann-Straße 13, 67071 Ludwigshafen am Rhein, Germany; ^4^Department of Orthopaedics and Trauma Surgery, Heidelberg University Hospital, Schlierbacher Landstrasse 200a, 69118 Heidelberg, Germany

## Abstract

Congenital unilateral hypertrophy of the plantar musculature is a rare condition, and to our knowledge, reports of only 14 cases have been previously published. As only one describes a concomitant orthopedic toe deformity, we report our case of abductor hallucis, flexor digitorum brevis, and abductor digiti minimi muscle hypertrophy in combination with hallux valgus and claw toe deformity as well as a laterally abducted fifth toe. Thus, this report presents the rare case of congenital hypertrophy of the plantar musculature associated with complex toe deformities. Moreover, the present article contains a detailed description of our surgical technique as well as a review of the current literature.

## 1. Introduction

Congenital unilateral hypertrophy of the plantar musculature is a rare condition of unknown incidence. In 1974, Jahss was the first to publish a description of a congenital hypertrophy of the quadratus plantae muscle, which he assessed as “pseudotumor of the foot” in a 4-year-old female [1]. Since then, nine more cases of isolated muscle hypertrophy have been reported [2–9]. Moreover, four authors have presented hypertrophy of multiple muscles [10–13]. However, only one of these 14 articles describes a concomitant orthopedic toe deformity or, more concretely, two hammer toes and no publication has formerly correlated this condition with elevated blood creatine kinase levels [13].

This article reports the unusual case of a 17-year-old man with this rare condition in combination with multiple toe deformities: hallux valgus, claw toe (second toe), and laterally abducted digitus quintus. To our knowledge, this atypical presentation of complex osseous and soft tissue deformities has not been previously published in the medical literature. Therefore, this case study is aimed at reporting a detailed description of the present patient and explains the rationale behind our chosen surgical procedure. Moreover, a review of the current literature related to congenital hypertrophy of the plantar musculature is presented to discuss its possible etiology as well as diagnostic and therapeutic regimen.

## 2. Case Report

**Figure 1:**
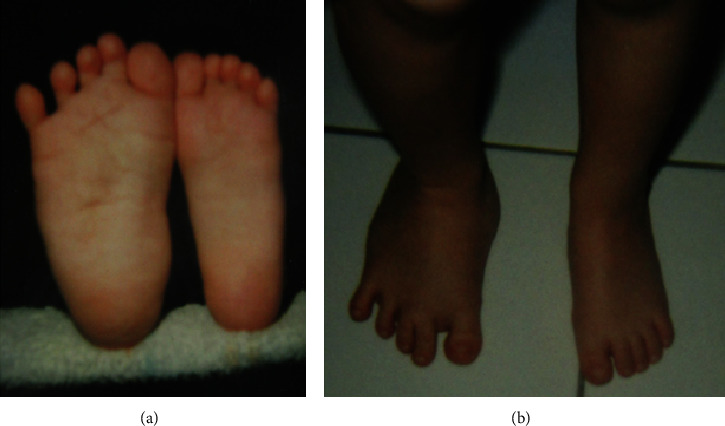
Photograph of the plantar (a) and dorsal (b) sides of the patient's feet as a child (2 years), as provided by the parents.

A 17-year-old man presented with painless hemihypertrophy of the right foot and calf and was treated at our institution between 10.2017 and 12.2019. The asymmetry of his feet, which has never been progressive, was first noticed at the time of his birth (Figure 1). Since then, the patient has been forced to wear a shoe on his right foot that is two sizes larger (European shoe size) than on his left foot. Moreover, an initial leg length discrepancy of 1 centimeter at the time of this birth increased to 1.5 centimeter. Therefore, he was provided with heightening orthotic insoles.

During maturity, his only physical complaints had been muscle cramps on the lateral aspect of his foot, which occasionally occurred after extensive activities. Apart from that, he experienced no difficulty in walking, running, or even playing football. However, since the age of 15, the patient suffered from typical hallux valgus deformity symptoms: pain over the medial eminence and local skin irritation.

Physical examination revealed a painless, soft mass on the medioplantar, plantar, and lateroplantar surface of the right foot. This soft tissue mass completely obliterated the plantar arch of the foot. Range of motion in the right upper limb's joints did not differ from the left side. Motor function, vascularization, and sensibility were normal. The patient exhibited clinical presentation of a pes transversoplanus in combination with a hallux valgus deformity, which showed a slight hallux valgus interphalangeus component. We also observed a claw deformity of the second toe and a digitus quintus with laterally abducted phalanges; however, neither were causing any clinical symptoms.

**Figure 2:**
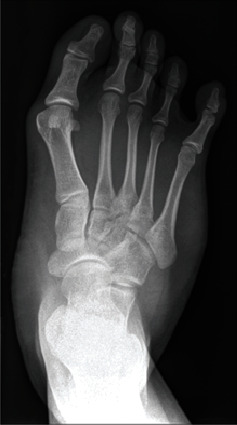
Preoperative anteroposterior radiograph.

**Figure 3:**
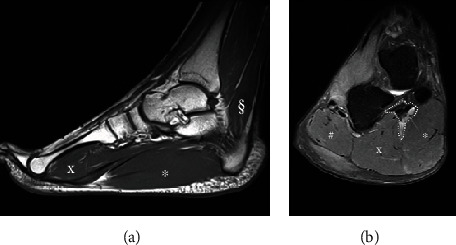
Sagittal (a) and coronal (b) MRI scans show hypertrophy of abductor hallucis (∗), flexor digitorum brevis (x), abductor digiti minimi (#), and accessorius soleus muscle (§).

Radiographs in two planes (posteroanterior and lateral) confirmed the clinical presentation (Figure 2). An intermetatarsal angle of 18.6° and a hallux valgus angle of 33.6° were measured. MRI imaging showed a constant hypertrophy of the short foot musculature with muscular-isointense nodular structures in the plantar subcutaneous fat tissue. Moreover, an accessory musculus soleus, with muscular portions ventral to the Achilles tendon, was shown to reach up to the calcaneus (Figure 3).

Routine laboratory testing revealed elevated blood creatine kinase levels: 306 units/liter (reference range: <190 units/liter).

### 2.1. Operative Technique

**Figure 4:**
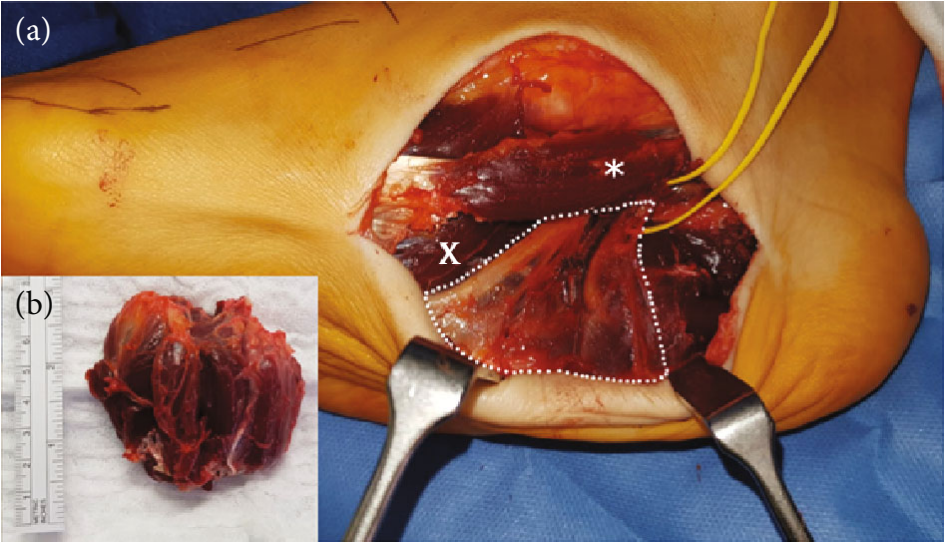
Intraoperative photograph (a) displaying the medial approach: reduction-plasty of abductor hallucis brevis muscle (∗); (b) shows the resected muscular portion), flexor digitorum brevis muscle in depth (x), and the delta-shaped neurovascular bundle (framed with white dots).

**Figure 5:**
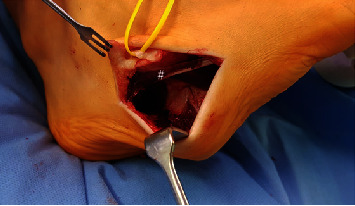
Intraoperative photograph demonstrating the lateral approach: reduction-plasty of the abductor digiti minimi muscle (#) and nervus suralis retracted with a vessel loop.

**Figure 6:**
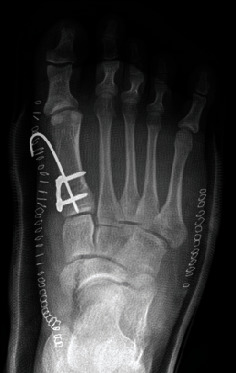
Postoperative posteroanterior radiograph, including plate and K-wire osteosynthesis.

**Figure 7:**
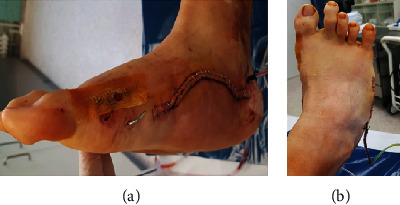
Postoperative photographs of the medial (a) and dorsal (b) side of the foot, displaying the wound with skin staples, a drain, a K-wire, fatty gauze covering the hallux metatarsophalangeal joint approach, and laterally abducted fifth toe.

The procedure was performed with the patient supine and under general anesthesia using an Esmarch's bandage for surgical hemostasis. First, a crescentic skin incision, approximately 10-centimeter-long, was made on the medial side of the right foot. After dissecting the abductor hallucis brevis muscle, the plantar hypertrophic portion of this muscle was resected with a cautery knife. The plantar portion of this muscle was insufficient, partly due to strong connective tissue fibers toward the subcutaneous fat tissue. Below this muscle, the neurovascular bundle appeared flattened in a delta-shaped manner in the intermuscular fascia (Figure 4). Next, the hypertrophic flexor digitorum brevis muscle was resected subtotally. Thus, the remaining abductor hallucis muscle lost physiological tension, which we addressed by using gathering stitches on its tendon. The next step included a lateral, straight, and approximately 6-centimeter-long approach to the hypertrophic abductor digiti minimi muscle, which was also freed and decreased in volume (Figure 5). The surgery then proceeded to correcting the hallux valgus deformity. After the soft tissue of the hallux metatarsophalangeal joint was released, plication of the medial part of the capsule and proximal osteotomy of the first metatarsal was performed [14]. For osteosynthesis, we chose an ankle-stable plate system and a temporary K-wire fixation (Figure 6). Following hemostasis, a drain was placed both in the medial and lateral wound cavity, and dermal closure was performed with skin staples (Figure 7).

**Figure 8:**
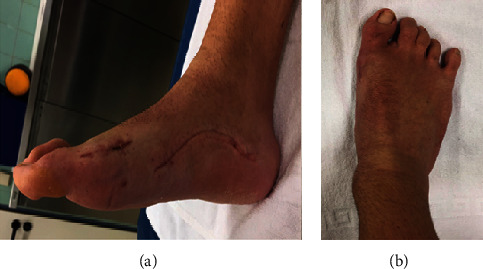
Photographs of the medial (a) and dorsal (b) side of the foot nine weeks postoperatively, displaying healed wounds, and corrected hallux valgus deformity, as well as an already significant decrease in volume of the plantar aspect of the foot.

Histological analysis of the resected tissue revealed skeletal muscle fiber hypertrophy as well as focal skeletal muscle atrophy, microfocal skeletal muscle necrosis, and local, chronic inflammation with interstitial fibrosis. Postoperatively, the foot was immobilized with a below-knee cast for six weeks, after which the K-wire was removed (Figure 8). Following, he was provided with a removable cast and we recommended partial stress for three weeks. After this period, he received orthotic insoles with medial arch support and a dynamic ankle-foot orthosis for night use and could move on to full stress. Finally, this led to resolution of his symptoms as well as clinical and radiological correction of his orthopedic toe deformities. Moreover, the blood creatine kinase levels declined to a normal range.

## 3. Discussion

Congenital isolated hypertrophy of the plantar musculature is a rare condition with only 14 cases previously described in the literature. As with other accessory tissue of the foot, e.g., polydactyly with additional digits consisting of soft tissue, no information on incidence or etiology is available [15]. Although all these cases exhibited hypertrophy of one isolated or multiple muscle bellies, it is questionable whether all of these cases have the same underlying pathophysiology. Hellwinkel et al. [13] summarized five common characteristics of congenital hypertrophy cases, including “soft tissue mass present from birth, nontender to palpation (may cause pain with activity), normal bony anatomy on radiographs, unilateral presentation, and generally nonprogressive.”

These characteristics have been reported among patients between four months and 33 years. Left and right feet are approximately equally affected. In reviewing the literature, we identified 14 cases of this condition, all of which occurred without any correlation to a congenital syndrome or disorder. We have decided to present cases affecting an isolated muscle (Table 1) separately from cases with hypertrophy of multiple muscle bellies (Table 2).

Table 1 includes 10 cases of hypertrophy of an isolated muscle; hypertrophy of the abductor hallucis muscle was the most frequently reported (six occurrences) [2–4, 7, 9]. The abductor hallucis muscle covers the retinaculum flexorum, which represents the medial margin of the tarsal tunnel. For this reason, hypertrophy can cause nerve entrapments, as reported by Boeren et al. [7] and Kurashige [9]. Moreover, Kurashige [9] published a review focusing on hypertrophy of the abductor hallucis and was able to present five cases of subsequent tarsal tunnel syndrome. Unfortunately, many of the articles providing information for this review do not specify whether the hypertrophy was congenital. Therefore, they are not eligible for our present review. Three cases included an isolated hypertrophy of the abductor digiti minimi, and the first report of this disorder was published by Jahss [1] about quadratus plantae muscle hypertrophy [1, 5, 6, 8]. The most common clinical complaints were problems with “normal” footwear, which presented even more often than pain of any level. Interestingly, nine out of ten cases are females between 4 and 20 years. Kurashige [9] hypothesizes that “increased body weight, increased activity duration, and footwear may affect this condition of young women.” Although he is not strictly reporting about congenital cases, we do not share this opinion, because congenital cases, like the one we report, exclude an extrinsic etiology by definition. Presuming that all cases in Tables 1 and 2 (i.e., isolated and multiple muscle hypertrophies) represent the same condition, we hypothesize a vascular genesis. We were able to detect an importantly increased rete venosum dorsalis pedis on the affected side compared to the contralateral side. Although these veins merely indicate the epifascial drainage status, we assume that an increased blood and nutrient supply could be a congenital, intrinsic trigger for this condition. Moreover, the present case is the first one in the literature which reports elevated creatine kinase blood levels in correlation with this condition. We assume that the compressive stress on the hypertrophic muscular mass while walking caused microfocal skeletal muscle necrosis (confirmed in our histological analysis) and, in further consequence, elevated creatine kinase blood levels.

Table 2 provides a more detailed overview of hypertrophy affecting multiple plantar muscles. The cases reported in the literature range from three affected muscles, as in the case of our patient, to all plantar muscles and even to all intrinsic muscles of the foot [10–13]. Gender distribution is more balanced within these cases (three women versus one man). The most common diagnostic pathway consisted of physical examination, radiographic bone evaluation, and MRI for soft tissue analysis, which revealed “muscular-isointense structures” in all cases. However, Hellwinkel et al.'s [13] characteristics of this condition include “generally nonprogressive,” which cannot be confirmed by a single MRI, but rather require a clinical follow-up. Moreover, ultrasound represents an alternative diagnostic tool, which is successfully used for assessing musculoskeletal conditions of the foot, e.g., hallux valgus deformity has already been correlated with reduced cross-sectional area and thickness of the abductor hallucis and flexor hallucis brevis as well as increased thickness of the plantar fascia [16]. The photopodogram method, a well-tried technique in diagnostics of foot deformities, can also be used for evaluating side differences and the progression of the plantar mass or for assessing the postoperative result. Using this method, López-López et al. have conducted surveys correlating the foot arch height and quality-of-life parameters [17, 18]. Boothroyd and Carty suggest that muscular biopsy is not required for histological verification in general but should definitely be considered if any other differential diagnosis, especially a malignant entity, is possible [19].

With respect to treatment options for this condition, we emphasize that surgical intervention should only be indicated if clinical symptoms cannot be handled with more conservative options. Particularly, resection of functional plantar musculature has several potential complications, such as motor imbalances causing secondary joint deformities, local paralysis due to nerve injury, necrosis due to compromised blood supply, and general wound healing and scar problems. In this context, Figure 5 clearly illustrates the adaptability of the neurovascular bundle because it is completely flattened in the intramuscular fascia without causing any clinical symptoms, such as nerve entrapment and hypoperfusion. Therefore, our indication for surgery was a symptomatic hallux valgus deformity, which we believed to result from muscular imbalance and unphysiological muscular traction caused by the condition.

In summary, our procedure is aimed at relieving the hallux valgus symptoms, correcting its triggering factor, and preventing the patient from further symptomatic foot deformities, while restoring—and not destroying—the physiological muscular function by partial resection. For this reason, we did not surgically address the asymptomatic claw deformity on the second toe or the laterally abducted fifth toe, assuming that these deformities will stop proceeding or even regress after removal of the presumed triggering factor.

## Figures and Tables

**Table 1:** Literature review of previous cases concerning congenital unilateral hypertrophy of an isolated plantar muscle.

Author	Age	Gender	Side	Clinical complaint	Muscles involved
Jahss [1]	4 years	Female	Left	Swelling of the plantar arch	QP
Ross and Lepow [2]	15 years	Female	Right	Pain with shoe wear	AH
Ringelman and Goldberg [3]	14 years	Female	Right	Footwear problem	AH
Torigoshi et al. [4]	16 years	Female	n/a	Footwear problem	AH
	10 months	Male	n/a	Foot enlargement	AH
Iconomou et al. [5]	20 years	Female	Left	Footwear problem	ADM
Raab et al. [6]	15 years	Female	Left	Footwear problem	ADM
Boeren et al. [7]	13 years	Female	Right	Radiating pain to the 1^st^ and 2^nd^ toe	AH
Schmauss et al. [8]	14 years	Female	Right	Footwear problem and pain during activity	ADM
Kurashige [9]	14 years	Female	Left	Pain in the morning	AH

QP = quadratus plantae muscle; AH = abductor hallucis muscle; ADM = abductor digiti minimi muscle; n/a = not applicable.

**Table 2:** Literature review of previous cases concerning congenital unilateral hypertrophy of multiple plantar muscles.

Author	Age	Gender	Side	Clinical complaint	Workup	Muscles involved	Treatment	Outcome
Estersohn et al. [10]	33 years	Female	Right	Pain/spasms of 4th and 5th toes, especially while exercising	Radiographs, biopsy	ADM, 3^rd^ and 4^th^ plantar interossei	ADM total resection	Resolution of symptoms
Sevin et al. [11]	18 years	Female	Left	Footwear problem	Radiographs, MRI	FDB, AH, FHB	AH, FHB total resection	Resolution of symptoms
Shiraishi et al. [12]	4 months	Male	Left	Foot enlargement	Radiographs, MRI	All plantar muscles	ADM, FDM, AH, FHB (medial head) total and FDB partial resection	No symptoms
Hellwinkel et al. [13]	12 years	Female	Right	Hammer toe deformity with crossover	Radiographs, MRI	All foot intrinsics	Hammer toe correction, 2^nd^ and 3^rd^ PIP fusion	Resolution of symptoms
Current case	20 years	Male	Right	Hallux valgus symptoms, cramps on the lateral side of the foot	Radiographs, MRI	AH, FDB, ADM	AH, FDB and ADM partial resection, MT1 osteotomy and MTP joint soft tissue release	Resolution of symptoms

ADM = abductor digiti minimi muscle; FDB = flexor digitorum brevis muscle; AH = abductor hallucis muscle; FHB = flexor hallucis brevis muscle; FDM = flexor digiti minimi muscle.
